# Editorial: AI as intelligent technology and agent to understand and be understood by human minds

**DOI:** 10.3389/fpsyg.2024.1461881

**Published:** 2024-10-10

**Authors:** Hao Chen, Xiaopeng Ren, Lingnan He, Junming Huang

**Affiliations:** ^1^Department of Social Psychology, School of Sociology, Nankai University, Tianjin, China; ^2^CAS Key Laboratory of Behavioral Science, Institute of Psychology, Chinese Academy of Sciences, Beijing, China; ^3^Department of Psychology, University of Chinese Academy of Sciences, Beijing, China; ^4^Department of Psychology, Sun Yat-sen University, Guangzhou, China; ^5^Center on Contemporary China, Princeton University, Princeton, NJ, United States

**Keywords:** health chatbots, human-AI collaboration, AI acceptance in organizations, environmental psychology, AI-ization of academic research, interpretable neural networks, ChatGPT, trustworthy AI

## 1 Introduction

The rapid evolution of artificial intelligence (AI) is profoundly reshaping our lives and work, demonstrating significant potential in areas such as industrial automation, business operations, academic research, and daily applications (Arin, 2023). However, the widespread use of AI has also raised concerns over privacy, algorithmic bias and transparency, and security risks, highlighting the need for ethical, safe, and socially responsible AI systems (Díaz-Rodríguez et al., 2023). Addressing these issues depends on how we further develop AI as a tool to better learn, fit, and align with human psychology and behavior, as well as how humans adapt to and better interact with AI as a social counterpart.

The six empirical and theorizing research papers collected under this Research Topic cover a range of areas or themes, including the acceptance of AI applications in personal health management, consumer behavior, and organizational behavior, the AI-ization of academic research, interpretable AI algorithms, and the comprehensive framework for trustworthy AI. These studies not only align with the objectives set by the Research Topic but also address the issues raised to varying degrees. They are expected to inspire future research to continue deepening and expanding in related fields.

## 2 Key findings and insights from the Research Topic

Within the realm of this Research Topic, encompassing both empirical and theoretical inquiries, whether it delves into the interpretability of AI algorithms or explores the acceptance and implementation of AI across various fields, the core issue consistently centers on how to cultivate trustworthy AI.

Zou et al. applied Innovation Resistance Theory (IRT) and the Prototype Willingness Model (PWM) to analyze public resistance to health chatbots, identifying functional and psychological barriers, along with negative prototype perceptions, as key resistance drivers. Their work advances understanding of resistance to emerging healthcare AI technologies.

Yue and Li explored how different human-AI collaboration modes and outcome expectations affect consumer evaluations and intentions through online experiments. They underlined the significance of responsibility attribution and algorithm transparency, noting a preference for AI-dominant products with positive expectations and AI-assisted ones with negative expectations. This research extends consumer behavior studies by including the human-AI interaction perspective, offering insights for better AI integration into products and services.

Fousiani et al.'s empirical study under our Research Topic on the interactive effects of competitive organizational climate and leadership power perception on employee AI acceptance revealed that a competitive environment fosters AI acceptance when leaders view power as a responsibility but hinders it when seen as an opportunity, enriching our understanding of organizational factors in AI acceptance and suggesting ways to enhance AI adoption in organizations.

Three additional theorizing research on trustworthy AI within our Research Topic deliver profound insights too. Liu and Xu outlined two interpretable neural network (INN) methodologies: Model Decomposition and Semantic INNs. The former decomposes traditional mathematical or physical models into learnable modules, making the computational process interpretable by translating it into network parameters. The latter provides semantic explanations for black-box networks through visualization, decision tree regularization, and semantic knowledge graphs. The two approaches, based on pre- or post-network-design respectively, aim to enhance the transparency and interpretability of deep neural networks, thus promoting humans's trust in them.

Yuan et al. focused on the AI-ization of academic research, discussing generative AI's role, like ChatGPT, in environmental psychology. They highlighted its potential to improve research by aiding in question formulation, theory modeling, and tool development, as well as streamlining data analysis and customizing communication. However, they stressed the importance of ethical considerations and the need for professional training and evaluation to ensure research quality and integrity.

Last but not least, as a summary of our Research Topic and its related research, Li et al. encapsulate the essence of building trustworthy AI systems by examining critical factors from a tripartite framework of trustor, trustee, and their interactive context. They highlight the importance of demographic characteristics, familiarity with AI, self-efficacy, and emotional experiences for human trustors, alongside the technical attributes of AI systems such as reliability and ethical alignment. Additionally, the influence of cultural and organizational contexts on trust dynamics is emphasized. This synthesis underscores the need for a holistic approach that integrates technical excellence with human-centric ethics to enhance AI acceptance.

## 3 Progress and prospects

The excellent papers above delve into developing trustworthy AI systems from various angles, including user acceptance, organizational environment, algorithm interpretability, the AI-ization of academic research and so on, offering rich perspectives and insights. Key factors affecting AI acceptance identified include functionality, psychological aspects, accountability, and transparency of algorithms. For example, Zou et al. highlights how overcoming functional and psychological barriers to health chatbots and improving their prototype perceptions can boost public acceptance. Additionally, organizational factors like leadership style significantly influence AI adoption; Fousiani et al. found that employees are more receptive to AI when leaders view power as a responsibility rather than an opportunity, underscoring the need for adaptive organizational cultures and management styles. Moreover, advancements in interpretable AI algorithms summarized by Liu and Xu, such as model decomposition and semantic neural networks, are crucial for making AI's decision-making processes more understandable and transparent, thus fostering trust among users.

Despite progress in the field, critical challenges remain. Firstly, deepening our understanding of human psychological expectations and trust mechanisms toward AI is essential. Current research often focuses on single dimensions, lacking a holistic view of the human-AI interaction process. Future studies should adopt an integrated theoretical framework to explore the interplay between trustors, trustees, and interaction contexts. Secondly, the question of designing more humane and ethically aligned AI systems is significant. Existing technological solutions struggle to fully address biases and safety concerns in AI. Researchers should refine interpretable AI algorithms and enhance assessments of AI systems' ethics and social impacts to align with human values. Lastly, improving organizational and societal-level AI governance is challenging, with most research focusing on the individual level and lacking systematic policy and institutional recommendations. Efforts should aim at establishing relevant regulations and standards, promoting stakeholder participation and collaboration, to foster healthy AI development.

In summary, building truly trustworthy AI requires collaborative efforts across technical, ethical, and governance dimensions from scholars, businesses, and governments. Only through such comprehensive efforts can AI become an intelligent technology and agent that is both understanding and understandable by humanity.

## References

[B1] Arin H. (2023). AI and the Human Experience: Embracing the Age of Intelligent Machines. San Diego: Holly Arin.

[B2] Díaz-Rodríguez N. Del Ser J. Coeckelbergh M. de Prado M. L. Herrera-Viedma E. Herrera F. (2023). Connecting the dots in trustworthy Artificial Intelligence: from AI principles, ethics, and key requirements to responsible AI systems and regulation. Inform. Fus. 99:101896. 10.1016/j.inffus.2023.101896

